# Data for praying mantis mitochondrial genomes and phylogenetic constructions within Mantodea

**DOI:** 10.1016/j.dib.2018.10.070

**Published:** 2018-10-25

**Authors:** Le-Ping Zhang, Dan-Na Yu, Kenneth B. Storey, Hong-Yi Cheng, Jia-Yong Zhang

**Affiliations:** a College of Chemistry and Life Science, Zhejiang Normal University, Jinhua 321004, Zhejiang Province, China; b Key Lab of Wildlife Biotechnology, Conservation and Utilization of Zhejiang Province, Zhejiang Normal University, Jinhua, Zhejiang Province, China; c Department of Biology, Carleton University, Ottawa, Ontario, Canada K1S 5B6

## Abstract

In this data article, we provide five datasets of mantis mitochondrial genomes: (1) PCG123: nucleotide sequences of 13 protein-coding genes including all codon positions; (2) PCG123R: nucleotide sequences of two rRNAs and 13 protein-coding genes including all codon positions; (3) PCG12: nucleotide sequences of 13 protein-coding genes without third codon positions; (4) PCG12R: nucleotide sequences of two rRNAs and 13 protein-coding genes without third codon positions, and (5) PCGAA: amino acid sequences of 13 protein-coding genes. These were used to construct phylogenetic relationships within Mantodea and the phylogenetic trees inferred from Bayesian analysis using two data sets (PCG12R, PCGAA) and Maximum Likelihood analysis using four data sets (PCG123, PCG12, PCG12R and PCGAA). We also provide initiation codon, termination codon, amino acid length and nucleotide diversity (Pi) of protein-coding genes among 27 mantises. The whole mitochondrial genomes of 27 praying mantises were submitted to GenBank with the accession numbers KY689112–KY689138.

**Specifications table**

**Table:** 

Subject area	Biology
More specific subject area	Phylogenetics; Mitochondrial Genomics
Type of data	Figure, text file, graph and table
How data was acquired	Sanger DNA sequencing
Data format	Phylogenetic trees are in figure format (.eps) and newick format (.nwk) and mitochondrial DNA sequence alignments are in paup format (.nexus).
Experimental factors	Total genomic DNA was extracted from leg muscle. DNA sequences were acquired by PCR and Sanger sequenced by Sangon Biotech Company.
Experimental features	Sequence fragments were assembled using DNASTAR Package v.6.0. Nucleotide sequences and amino acids sequences of 13 protein-coding genes were used to construct phylogenetic trees by MrBayes 3.2 and RAxML 8.2.0.
Data source location	Specimens were collected from Africa, China, Indonesia and Malaysia.
Data accessibility	Five datasets (.nexus files) used to construct phylogenetic trees and newick tree files (.nwk files) are provided here. The whole mitochondrial genomes are available in GenBank with the accession numbers KY689112–KY689138.

**Value of the data**

• The mitochondrial genomes of praying mantises are good models for future study of gene rearrangements and gene duplications.
• The primer strategy used to amplify the mantis mitochondrial genomes could be widely used for other insect mitochondrial genomes and this strategy can greatly reduce the experimental workload needed to acquire whole genome sequences.
• The phylogenetic relationships within Mantodea inferred from BI analyses using 2 data sets (PCG12R, PCGAA) and ML analysis using four data sets (PCG123, PCG12, PCG12R and PCGAA) show a few differences with the phylogenetic relationships reported in the main text, which is worthy of further discussions.
• The data presented here will be useful to solve the phylogenetic relationships within Mantodea.

## 1 Data

The data presented here originate from a study of higher tRNA gene duplication in the mitogenomes of praying mantises (Dictyoptera, Mantodea) and the phylogeny within Mantodea [1], including genome statistics and phylogenetic trees. The monophyly of Mantodea is supported [2], [3], [4], [5], [6] whereas the phylogenetic relationships within Mantodea are under suspicion especially in two large families: Mantidae and Hymenopodidae [7], [8]. Our study supported the monophyly of Liturgusidae and Iridopterygidae and the paraphyly of Hymenopodidae, Mantidae and Tarachodidae [1].

**Fig. 1:**
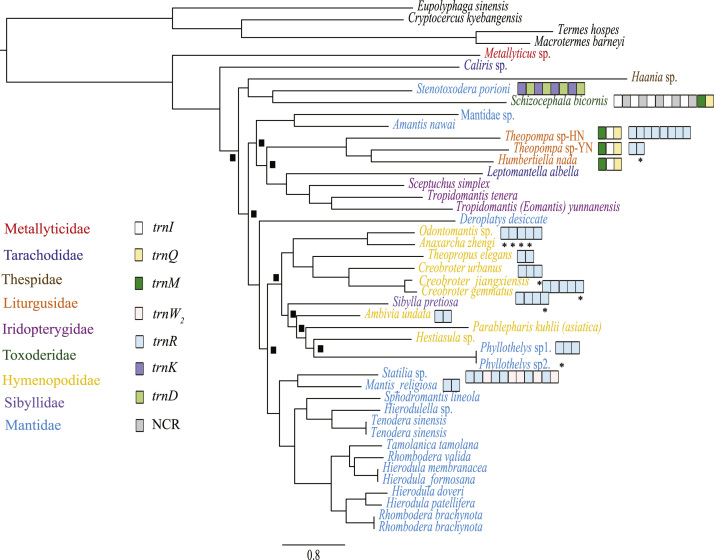
Phylogenetic relationships of Mantodea analyzed with ML methods using the dataset 13PCG. At each node, the black box indicates BP < 75. Other small boxes represent tRNA genes and the box labeled with an asterisk represents a pseudogene.

**Fig. 2:**
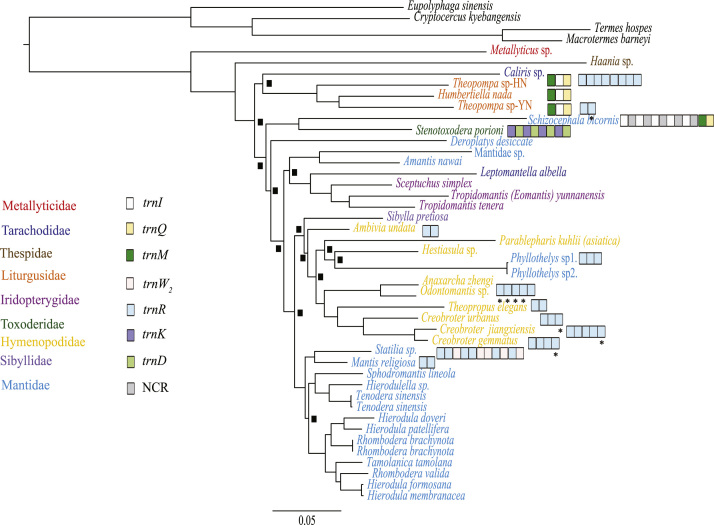
Phylogenetic relationships of Mantodea analyzed with ML methods using the dataset 13PCG12. At each node, the black box indicates BP < 75. Other small boxes represent tRNA genes and the box labeled with an asterisk represents a pseudogene.

**Fig. 3:**
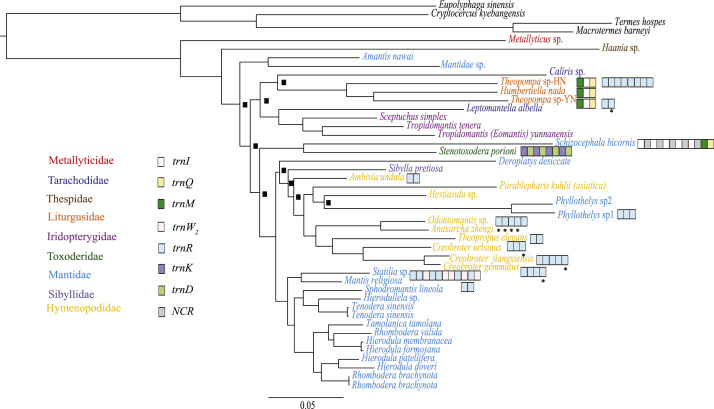
Phylogenetic relationships of Mantodea analyzed with ML methods using the dataset 13PCG12R. At each node, the black box indicates BP < 0.75. Other small boxes represent tRNA genes and the box labeled with an asterisk represents a pseudogene.

**Fig. 4:**
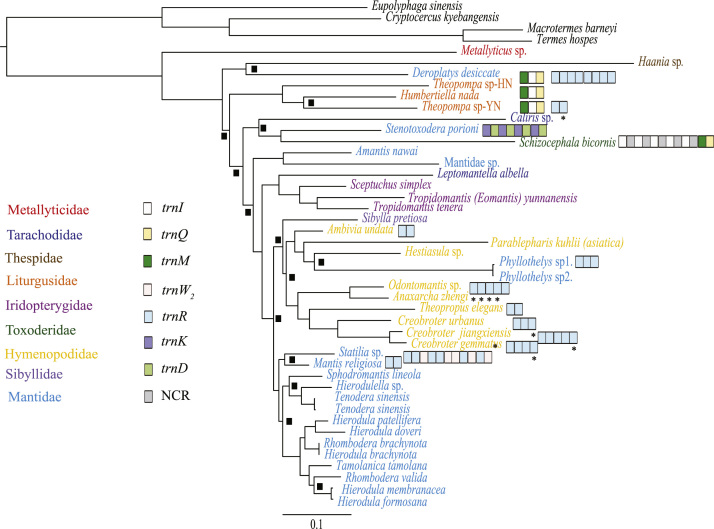
Phylogenetic relationships of Mantodea analyzed with ML methods using the dataset 13PCGAA. At each node, the black box indicates BP < 0.75. Other small boxes represent tRNA genes and the box labeled with an asterisk represents a pseudogene.

**Fig. 5:**
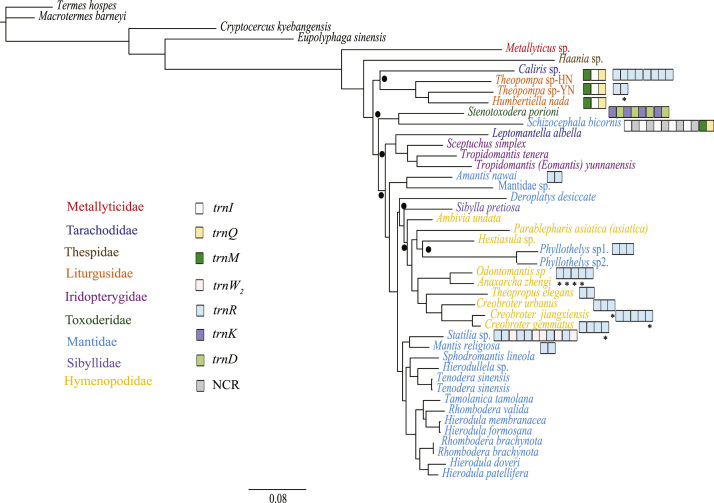
Phylogenetic relationships of Mantodea analyzed with BI methods using the dataset 13PCG12R. At each node, the black box indicates PP < 0.95. Other small boxes represent tRNA genes and the box labeled with an asterisk represents a pseudogene.

**Fig. 6:**
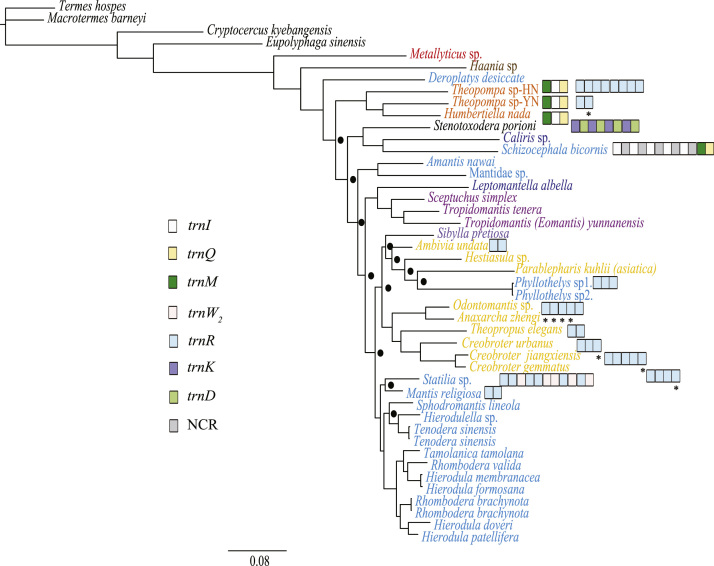
Phylogenetic relationships of Mantodea analyzed with BI methods using the dataset 13PCGAA. At each node, the black box indicates PP < 0.95. Other small boxes represent tRNA genes and the box labeled with an asterisk represents a pseudogene.

**Fig. 7:**
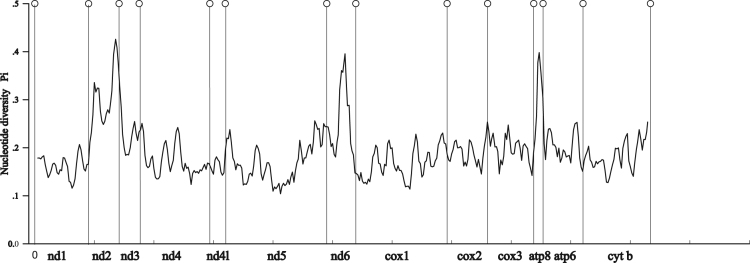
Nucleotide diversity of protein-coding genes among 27 mantises.

Five data sets were used to perform Maximum Likelihood analysis (ML) and Bayesian Inference (BI): (1) PCG123: 13 PCGs including all codon positions; (2) PCG123R: two rRNAs and 13 PCGs including all codon positions; (3) PCG12: 13 PCGs without third codon positions; (4) PCG12R: two rRNAs and 13 PCGs without third codon positions, and (5) PCGAA: amino acid sequences of 13 PCGs. The phylogenetic relationships inferred from BI analyses using 3 data sets (PCG123, PCG123R, PCG12) and ML analyses using the data set PCG123R shared the same topologies. Hence, we illustrated nodal supports from the four analyses together, which are data provided in the main text [1]. Here, we present the phylogenetic relationships inferred from BI analyses using two data sets (PCG12R, PCGAA) and ML analyses using four data sets (PCG123, PCG12, PCG12R and PCGAA) (Fig. 1, Fig. 2, Fig. 3, Fig. 4, Fig. 5, Fig. 6). These phylogenetic trees can be compared to trees presented in the main text. The sequences of the five datasets are also provided. The initiation codon, termination codon and amino acids length of protein-coding genes are compared among 27 mantises (Table 1) and the nucleotide diversity of protein-coding genes are calculated (Fig. 7).

**Table 1:** Initiation codon (I codon), termination codon (T codon) and amino acids length of protein-coding genes among 27 mantises.

**Species**		***nd2***	***cox1***	***cox2***	***atp8***	***atp6***	***cox3***	***nd3***	***nd5***	***nd4***	***nd4l***	***nd6***	***cytb***	***nd1***
*Ambivia undata* (Hymenopodidae)	No.	342	511	229	52	225	263	117	574	445	93	167	378	310
	I codon	ATG	TTG	ATA	ATC	ATA	ATA	ATC	ATG	ATG	ATG	ATT	ATG	ATG
	T codon	TAA	TAA	T-	TAA	TAA	TAA	TAA	T-	TAA	TAA	TAA	TAA	TAA
*Hestiasula* sp. (Hymenopodidae)	No.	342	511	230	52	225	263	117	575	445	93	167	377	310
	I codon	ATG	TTG	ATT	ATT	ATA	ATA	ATT	ATG	ATG	ATG	ATT	ATG	ATG
	T codon	TAA	TAA	TAA	TAA	TAA	TAA	TAA	T-	TAA	TAA	TAA	TAA	TAA
*Odontomantis* sp. (Hymenopodidae)	No.	342	512	229	52	225	262	117	573	445	93	167	378	313
	I codon	ATG	TTG	ATG	ATC	ATA	ATG	ATT	ATG	ATG	ATG	ATT	ATG	ATT
	T codon	TAA	TAA	T-	TAA	TAA	TAA	TAA	TAA	TAA	TAA	TAA	TAA	TAA
*Parablepharis kuhlii asiatica* (Hymenopodidae)	No.	345	514	229	52	224	262	117	575	439	93	167	378	314
	I codon	ATG	TTG	ATG	ATT	ATA	ATG	ATT	ATG	ATT	ATG	ATG	ATG	ATA
	T codon	TAA	TAA	TAA	TAA	TAA	TAA	TAA	T-	TAG	TAA	TAA	TAA	TAA
*Creobroter jiangxiensis* (Hymenopodidae)	No.	342	513	229	52	225	262	117	575	445	93	167	377	310
	I codon	ATG	ATC	ATG	ATT	ATA	ATG	ATT	ATG	ATG	ATG	ATT	ATG	ATG
	T codon	TAA	TAA	T-	TAA	TAA	TAA	TAA	T-	TAA	TAA	TAA	TAA	TAA
*Creobroter urbanus* (Hymenopodidae)	No.	342	512	229	52	225	262	117	574	445	93	167	378	310
	I codon	ATG	TTA	ATG	ATT	ATA	ATG	ATT	GTG	ATG	ATG	ATT	ATA	ATG
	T codon	TAA	TAA	T-	TAA	TAA	TAA	TAA	T-	TAA	TAA	TAA	TAG	TAA
*Theopropus elegans* (Hymenopodidae)	No.	342	511	229	52	225	262	117	574	445	93	165	377	310
	I codon	ATG	ATG	ATG	ATT	ATA	ATG	ATT	GTG	GTG	ATG	ATT	ATG	ATG
	T codon	TAA	TAA	TAG	TAA	TAA	TAA	TAA	T-	TAA	TAA	TAA	TAA	TAA
*Sceptuchus simplex* (Iridopterygidae)	No.	342	511	228	52	225	262	117	574	445	93	167	377	310
	I codon	ATG	TTG	ATG	ATT	ATA	ATG	ATT	GTG	ATG	ATG	ATT	ATG	ATG
	T codon	TAA	TAA	T-	TAA	TAA	T-	TAA	T-	TAA	TAA	TAA	TAA	TAA
*Eomantis yunnanensis* (Iridopterygidae)	No.	342	511	227	52	224	262	117	574	445	95	167	378	311
	I codon	ATG	TTG	ATG	ATT	ATA	ATG	ATT	ATG	ATG	ATA	ATT	ATG	ATG
	T codon	TAG	TAA	TAA	TAA	TAA	TAA	TAA	T-	TAA	TAA	TAA	TAA	TAA
*Tropidomantis tenera* (Iridopterygidae)	No.	342	511	228	53	225	262	117	573	445	93	167	378	311
	I codon	ATG	CTG	ATG	ATT	ATA	ATG	ATC	GTG	ATG	ATG	ATT	ATG	ATG
	T codon	TAA	TAA	T-	TAA	TAA	TAA	TAG	T-	TAA	TAA	TAA	TAA	TAA
*Amantis nawai* (Mantidae)	No.	342	511	228	52	225	262	117	573	445	93	165	377	311
	I codon	ATG	CTG	ATG	ATA	ATA	ATG	ATA	GTG	ATG	ATG	ATT	ATG	ATG
	T codon	TAA	TAA	T-	TAA	TAA	TAA	TAA	T-	TAA	TAA	TAA	TAA	TAA
*Tenodera sinensi* (Mantidae)	No.	342	512	230	52	225	263	117	574	445	93	167	378	311
	I codon	ATG	ATT	ATC	ATC	ATA	ATA	ATT	GTG	ATG	ATG	ATT	ATG	ATG
	T codon	TAA	TAA	T-	TAA	TAA	TAA	TAA	T-	TAA	TAA	TAA	TAA	TAA
*Sphodromantis lineola* (Mantidae)	No.	342	511	228	52	225	263	117	574	445	93	167	378	311
	I codon	ATG	CTG	ATG	ATC	ATA	ATA	ATC	ATG	ATG	ATG	ATT	ATG	ATG
	T codon	TAA	TAA	T-	TAA	TAA	TAA	TAA	T-	TAA	TAA	TAA	TAA	TAA
*Hierodulella* sp. (Mantidae)	No.	342	511	228	52	225	263	117	570	445	93	167	378	311
	I codon	ATG	TTG	ATG	ATT	ATA	ATA	ATT	GTG	ATG	ATG	ATT	ATG	ATG
	T codon	TAA	TAA	T-	TAA	TAA	TAA	TAA	T-	TAA	TAA	TAA	TAA	TAA
*Rhombodera brachynota* (Mantidae)	No.	342	512	228	52	225	263	117	574	445	93	167	378	311
	I codon	ATG	ATA	ATG	ATT	ATA	ATA	ATT	GTG	ATG	ATG	ATT	ATG	ATG
	T codon	TAA	TAA	T-	TAA	TAA	TAA	TAA	T-	TAA	TAA	TAA	TAA	TAA
*Hierodula chinensis* (Mantidae)	No.	342	511	226	52	225	262	117	574	445	93	167	377	311
	I codon	ATG	CTG	ATG	ATT	ATA	ATG	ATT	ATG	ATG	ATG	ATT	ATG	ATG
	T codon	TAA	TAA	T-	TAA	TAA	TAA	TAA	T-	TAA	TAA	TAA	TAA	TAA
*Hierodula membranacea* (Mantidae)	No.	342	513	228	52	225	263	117	574	445	93	167	378	311
	I codon	ATT	ATT	ATG	ATC	ATA	ATA	ATT	ATG	ATG	ATG	ATA	ATG	ATG
	T codon	TAA	TAA	T-	TAA	TAA	TAA	TAA	T-	TAA	TAA	TAA	TAA	TAA
*Deroplatys desiccate* (Mantidae)	No.	343	511	227	53	226	262	117	573	445	93	171	377	311
	I codon	ATG	TTG	ATG	ATT	ATA	ATG	ATA	ATG	ATG	ATG	ATA	ATG	ATG
	T codon	TAA	TAA	TAA	TAA	TAA	TAA	TAA	T-	TAA	TAA	TAA	TAA	TAA
*Phyllothelys* sp1. (Mantidae)	No.	343	512	228	52	225	262	117	574	445	94	167	377	310
	I codon	ATG	ATC	ATG	ATT	ATA	ATG	ATT	ATG	ATG	ATG	ATT	ATG	ATG
	T codon	TAA	TAA	TAA	TAA	TAG	TAA	TAA	T-	TAA	TAA	TAA	TAA	TAA
*Phyllothelys* sp2. (Mantidae)	No.	343	512	228	52	225	262	117	574	445	94	167	377	310
	I codon	ATG	ATC	ATG	ATT	ATA	ATG	ATT	ATG	ATG	ATG	ATT	ATG	ATG
	T codon	TAA	TAA	T-	TAA	TAG	TAA	TAA	TAA	TAA	TAA	TAA	TAA	TAA
*Schizocephala bicornis* (Mantidae)	No.	340	511	231	53	225	261	117	574	445	93	167	378	311
	I codon	ATG	TTG	ATT	ATT	ATA	ATG	ATT	ATG	GTG	ATG	ATT	ATG	ATA
	T codon	TAA	TAA	TAA	TAA	TAA	T-	TAA	T-	TAG	TAA	TAA	TAA	TAA
*Mantidae* sp. (Mantidae)	No.	342	511	239	53	225	264	117	574	445	93	167	379	312
	I codon	ATG	TTG	ATA	ATC	ATA	ATG	ATT	ATT	GTG	ATG	ATT	ATG	ATA
	T codon	TAA	TAA	TAA	TAA	TAA	TAA	TAA	T-	TAA	TAA	TAA	TAA	TAA
*Metallyticus* sp. (Metallyticidae)	No.		513	230	52	226	261	118	575	449	93	169	376	311
	I codon		TTG	ATG	ATC	ATT	ATG	ATT	GTT	ATA	ATG	ATC	ATG	ATG
	T codon	TAA	TAA	T-	TAA	TAA	TAA	TAA	T-	TAA	TAA	TAA	TAG	TAA
*Sibylla pretiosa* (Sibyllidae)	No.	342	513	228	52	225	263	117	574	445	93	168	378	310
	I codon	ATG	TTA	ATG	ATT	ATA	ATA	ATT	ATG	ATG	ATG	ATT	ATG	ATG
	T codon	TAA	TAA	T-	TAG	TAA	TAA	TAA	T-	TAA	TAA	TAA	TAA	TAA
*Caliris* sp. (Tarachodidae)	No.	342	511	228	52	225	262	118	574	444	93	168	378	313
	I codon	ATG	GTG	ATG	ATT	ATA	ATG	ATA	ATG	ATG	ATG	ATG	ATG	ATA
	T codon	TAA	TAA	TAA	TAA	TAA	TAA	TAA	T-	T-	TAA	TAA	TAA	TAG
*Haania* sp. (Thespidae)	No.		511	228	52	223	260	117	572	443	94	166	377	311
	I codon		TTG	ATG	ATA	ATA	ATA	ATC	GTG	ATG	ATA	ATT	ATG	ATG
	T codon	T-	TAA	T-	TAA	TAA	T-	TAA	T-	TAA	TAA	TAA	TAG	TAA
*Stenotoxodera porioni* (Toxoderidae)	No.	343	512	226	52	225	262	117	575	445	93	167	378	311
	I codon	ATG	TTA	ATG	ATC	ATA	ATG	ATG	GTG	ATG	ATG	ATC	ATG	ATG
	T codon	TAA	TAA	TAA	TAA	TAA	T-	TAA	T-	TAA	TAA	TAA	TAA	TAA

## 2 Experimental design, materials and methods

**Fig. 8:**
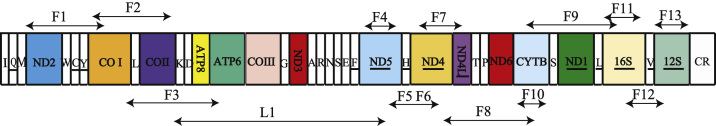
The primer strategy. Double-headed arrows indicate the location of the fragment amplified by PCR with each pair of primers. See Table 1 in the main text [1] for the primer DNA sequences associated with each fragment.

Our routine experimental approach was as follows: acquisition of the whole mitochondrial genomes of 27 mantises using total DNA extraction, PCR and sequencing; sequence analyses including assembly, annotation and alignment; and construction of phylogenetic relationships. The primer strategy is shown in Fig. 8 and primer sequences are given in the main text [1]. Five data sets (PCG123, PCG123R, PCG12, PCG12R and PCGAA) of 46 samples including 15 previously sequenced mantis mitogenomes [9], [10], [11], [12], [13] were used along with the mitogenomes of two cockroaches [14] and two termites [15], [16] as outgroups. ML and BI analyses were implemented in RAxML 8.2.0 [17] and MrBayes 3.2 [18], respectively. PartitionFinder 1.1.1 [19] was used to infer the optimal partitioning strategy and choose the best model. The nucleotide diversity of protein-coding genes among 27 mantises was calculated by DnaSP v5 [20]. A more detailed method and routine are provided in the main text [1].

## Appendix A Supplementary material

Supplementary_file1. The dataset PCG123: nucleotide sequences of 13 protein-coding genes including all codon positions.

.

Supplementary_file2. The dataset PCG123R: nucleotide sequences of two rRNAs and 13 protein-coding genes including all codon positions.

.

Supplementary_file3. The dataset PCG12: nucleotide sequences of 13 protein-coding genes without third codon positions.

.

Supplementary_file4. The dataset PCG12R: nucleotide sequences of two rRNAs and 13 protein-coding genes without third codon positions.

.

Supplementary_file5. The dataset PCGAA: amino acid sequences of 13 protein-coding genes.

.

Supplementary_file6. Phylogenetic trees in newick format conducted by ML methods using the dataset 13PCG.

.

Supplementary_file7. Phylogenetic trees in newick format conducted by ML methods using the dataset 13PCG12.

.

Supplementary_file8. Phylogenetic trees in newick format conducted by ML methods using the dataset 13PCG 12R.

.

Supplementary_file9. Phylogenetic trees in newick format conducted by ML methods using the dataset 13PCGAA.

.

Supplementary_file10. Phylogenetic trees in newick format conducted by BI methods using the dataset 13PCG12R.

.

Supplementary_file11. Phylogenetic trees in newick format conducted by BI methods using the dataset 13PCGAA.
