# Supplementary material for: Data for praying mantis mitochondrial genomes and phylogenetic constructions within Mantodea
Source: Data Brief. 2018 Oct 25;21:1277–85. doi: 10.1016/j.dib.2018.10.070 (PMC6230978; doi:10.1016/j.dib.2018.10.070)
Supplement: Supplementary file 1 — Supplementary material [file mmc1.docx]

**Statement of Conflict of interest**

The authors declare that the research was conducted in the absence of any commercial or financial relationships that could be construed as a potential conflict of interest.
